# Influenza and pneumococcal vaccine uptake among nursing home residents in Nottingham, England: a postal questionnaire survey

**DOI:** 10.1186/1471-2318-8-11

**Published:** 2008-05-16

**Authors:** Amir Shroufi, Joanna Copping, Roberto Vivancos, Richard CB Slack

**Affiliations:** 1 Norfolk PCT, St Andrew's House, St Andrew's Business Park, Northside, Norwich, NR7 0HT, UK; 2 Camden PCT, St Pancras Hospital, 4 St Pancras Way, London, NW1 0PE, UK; 3 School of Medicine, Health Policy & Practice' University of East Anglia Norwich, NR4 7TJ, UK; 4 East Midlands Health Protection Unit (North), Pleasley Vale Business Park, Mill 3, Floor 3, Pleasley, Mansfield, NG19 8RL, UK

## Abstract

**Background:**

Previous studies have shown influenza vaccine uptake in UK nursing home residents to be low. Very little information exists regarding the uptake of pneumococcal vaccine in this population. The formulation of policies relating to the vaccination of residents has been proposed as a simple step that may help improve vaccine uptake in care homes.

**Methods:**

A postal questionnaire was sent to matrons of all care homes with nursing within the Greater Nottingham area in January 2006. Non respondents were followed up with up to 3 phone calls.

**Results:**

30% (16/53) of respondents reported having a policy addressing influenza vaccination and 15% (8/53) had a policy addressing pneumococcal vaccination. Seasonal influenza vaccine coverage in care homes with a vaccination policy was 87% compared with 84% in care homes without a policy (p = 0.47). The uptake of pneumococcal vaccination was found to be low, particularly in care homes with no vaccination policy. Coverage was 60% and 32% in care homes with and without a vaccination policy respectively (p = 0.06). This result was found to be statistically significant on multivariate analysis (p = 0.03, R = 0.46)

**Conclusion:**

The uptake of influenza vaccine among care home residents in the Nottingham region is relatively high, although pneumococcal vaccine uptake is low. This study shows that there is an association between pneumococcal vaccine uptake and the existence of a vaccination policy in care homes, and highlights that few care homes have vaccination policies in place.

## Background

Care homes for the elderly facilitate rapid influenza spread and provide a suitable environment for outbreaks to occur [1,2]. Most care home residents are elderly and are at increased risk of severe complications from influenza [3]. Outbreaks of influenza in this setting may be associated with significant mortality [2]. In the UK all people over 65 are recommended to receive influenza vaccine as are all people in residential care homes [4]. Studies in UK care homes in the last decade have reported vaccine uptakes of 81–85% [3,5], which is below the level required for herd immunity. Inadequate policies and practices relating to influenza vaccine are one reason implicated in adversely affecting vaccine uptake in care homes [1,4].

*Streptococcus pneumoniae *(Pneumococcus) is the commonest cause of community acquired pneumonia and a common cause of pneumonia and bacteraemia, with rates highest in infants and the elderly. Uptake of pneumococcal vaccine in care homes has previously been very low, with a 2001 study in the UK describing coverage in nursing home residents of 11% [5]. In 2003 a new pneumococcal vaccination programme was phased in for older people, and since 2005 all people aged 65 years and over have been recommended to receive a dose of pneumococcal polysaccharide vaccine [6].

The Nottingham area includes a central conurbation of approximately 300,000 people, with an additional 350,000 people living in the surrounding suburban areas. This population is served by 58 care homes with nursing, 21 of which are located within the city's central conurbation. The term care home with nursing is used to describe facilities which provide nursing supervision and limited medical care to residents, and includes dual registered homes. According to the most recent census, 91% of residents of care homes with nursing in Nottingham are 65 years old or over.

We aimed to ascertain current uptake of influenza and pneumococcal vaccines among residents of care homes with nursing in Nottingham and assess whether having a vaccination policy in place influenced vaccine uptake.

## Methods

We conducted a questionnaire survey in January and February 2006. A postal questionnaire which was piloted in 5 randomly selected homes, was sent to matrons of all 58 of the care homes with nursing in the Nottingham area. Information was requested about uptake of influenza and pneumococcal vaccines among residents, number of general practitioner (GP) practices covering each home, vaccination policies and the approach to consent for vaccination. Questionnaires were coded to allow non-respondents to be followed up and information was treated confidentially. Non-respondents were followed up with a reminder letter, and up to 3 phone calls. We consulted the local ethics committee who advised that as the primary objective of our survey was to measure vaccine uptake ethical consent would not be required.

### Statistical analysis

We calculated Pearson's correlation coefficient to assess the association between the uptakes of both vaccines. An independent sample t-test was used to compare mean vaccine uptake among homes with relevant characteristics of the home (e.g. having vaccination register), the Mann-Whitney U test was used for ordinal variables (number of GPs and residents per home). We used a stepwise multiple linear regressing model, adding variables with a p-value less than 0.2, to adjust for potentially confounding variables upon the relationship between policy and vaccine uptake.

## Results

52 care homes returned a completed questionnaire, representing a response rate of 90%, with a total population of 1,759 residents. Adequate information on seasonal influenza and pneumococcal vaccine uptake was provided by 87% and 75% of care homes respectively, thus vaccine coverage was calculated on the appropriate denominators. The average number of residents per home was 34, and ranged from 14–89. Most of the residents, 87% (1579/1759) were over the age of 65. 71% (37/52) of care homes reported having one or more residents requiring residential care only. 30% (16/52) of homes reported having one or more resident under the age of 65.

### Vaccination uptake

The reported uptake of influenza and pneumococcal vaccine among the residents of care homes studied was 84% (26%–100%, SD = 0.16), and 36% (0%–100%, SD = 0.36) respectively. There was a positive correlation between homes with good influenza vaccine uptake and those with a good pneumococcal vaccine uptake, Pearsons correlation 0.35 (p = 0.03).

### Number of GPs covering care home

Care homes were served by a median of 5 GP practices (range 1–14). There was no statistical difference in uptake of influenza vaccine among care homes covered by less than 5 GP practices compared with care homes covered by more than 5 practices. Pneuemococcal vaccine uptake was higher in care homes which were covered by more than 5 practices.

### Vaccine administration

In 67% (35/52) of care homes the practice nurse provided vaccinations for most residents. In 17% (9/52) of care homes the home's own staff provided most vaccinations to residents, in 8% (4/52%) of care homes GPs provided most vaccinations and in one care home a health visitor was said to give most residents' vaccinations.

### Consent

79% (46/58) of care homes said they would always obtain family consent where a resident could not give informed consent, 3% (2/58) said they would vaccinate without expressed consent and the remainder did not respond to this question.

### Record keeping

Of care homes responding to our questionnaire, 96% (50/52), said that they would always record if 'flu vaccine was given in the patients' notes. One care home said they would not always do this and one did not respond to this question. Only 37% (19/52) of care homes said that they kept a seperate register of patients who had been given influenza vaccine, which was distinct from the patients' notes. 83% (43/52) of respondents said they would record pneumococcal vaccination in residents' notes and only 33% (17/52) of homes said they kept a register for recording residents' pneumococcal vaccination status.

### Care Home Policies on seasonal influenza and pneumococcal vaccination

30% of care homes (16/52), had a written policy on influenza. Vaccine uptake was not significantly different between care homes with and without a vaccination policy in place (p = 0.47), see table I. Only 15% (8/52) of care homes reported having a written policy on pneumococcal vaccine. Of those homes that did have a policy on pneumococcal vaccine, uptake was 60% compared to 34% among care homes that did not have such a policy (p = 0.06). After multivariate analysis, having a policy for pneumococcal vaccination remains independently associated with vaccine uptake (p = 0.03, R = 0.46).

**Table 1:** Influenza and pneumococcal vaccine uptake according to characteristics of care home.

		influenza vaccination			pneumococcal vaccination		
		Mean uptake (SD)	p-value	adjusted p-value†	Mean uptake (SD)	p-value	adjusted p-value†
Vaccination policy	No	84% (0.14)	0.47*	-	32% (0.32)	0.06*	0.03
	Yes	87% (0.15)			60% (0.46)		
Vaccine register	No	86% (0.18)	0.26*	-	35% (0.35)	0.68*	-
	Yes	80% (0.13)			40% (0.39)		
Vaccination recorded in residents' notes	No	84% (0.16)	0.99*	-	15% (0.36)	0.16*	-
	Yes	84% (0.16)			5% (0.31)		
Residents per home	<30	86% (0.13)	0.59^§^	-	29% (0.31)	0.17^§^	-
	≥ 30	82% (0.19)			46% (0.41)		
GPs per home	1–5	83% (0.17)	0.79^§^	-	25% (0.3)	0.03^§^	-
	>5	85% (0.16)			50% (0.39)		

Analysis used: * Independent samples t-test; §Mann-Whitney U test; † Multivariable linear regression using variables significant at a p < 0.2 level

## Discussion

### Main findings of this study

Our results show that uptake of influenza vaccine among care homes with nursing in the Nottingham area is relatively high at 84%. The uptake of pneumococcal vaccine in the same care homes is however much lower. Moreover, our study suggests that homes with a policy for pneumococcal vaccination have higher uptake of the vaccine.

For influenza, the level of uptake in Nottingham's care homes is consistent with previous studies among the UK care home population which report coverage of 81–85% [3,5,7], and represents a considerable improvement from the 39.6% uptake reported in Nottingham's care homes in 1992–1993 [8]. It is reassuring that the reported influenza vaccine shortage of 2005/6 due to enhanced demand as a result of increased public awareness of pandemic influenza does not appear to have adversely affected uptake, in our study population or nationally [9]. The upward trends observed in vaccine coverage are encouraging [10], and have no doubt have been helped by financial incentives to GPs through the Quality and Outcomes Framework and locally enhanced service payments. Nevertheless further improvement would promote herd immunity amongst this vulnerable population, where rapid spread is likely to follow introduction of infection, and lead to high morbidity and mortality[9].

As found by previous studies pneumococcal vaccination rates amongst our study population were low [5,11]. Although a number of studies have made recommendations for improving vaccine uptake in care homes, including joint policies between GPs and care homes [8], organised vaccine delivery strategies [5], and developing guidelines for care homes [12], no UK intervention studies to increase pneumococcal vaccine uptake in care homes were identified from the published literature. One study in Trent found that educational outreach visits to 15 general practices were associated with a significant increase in pneumococcal, but not influenza vaccine uptake amongst high risk groups compared to controls. Whether this effect was sustained beyond the 6 month study period is unknown [11]. A study of 133 long term care facilities in North America used a number of interventions including 'standing orders' (whereby nurses or pharmacists are authorised to administer vaccinations according to a pre-approved protocol), to increase pneumococcal vaccination rates from 40% to 75% [13]. Likewise, in Canada a pharmacist-centred standing orders intervention increased pneumococcal vaccine uptake among residents in the two care homes studied from 4.2% to 83% and from 1.9% to 83% [14]. As a result, the Centres for Disease Control and Prevention now recommend that standing orders be used in all long term care facilities [15]. In view of the fact that Nottingham care home residents appear to be vaccinated by practice staff rather than nursing home staff, it is unclear whether these benefits are generalisable to the UK.

As would perhaps be expected from previous North American research [16], the care homes in this study which had written policies for vaccination, had a higher vaccine uptake than homes which did not have such policies in place. In the case of pneumococcal vaccine this association was statistically significant on multivariate analysis. This may be a reflection of the quality of the care home and the importance placed on infection control procedures, equally it may be that policy directly affects uptake [17].

Smaller homes had higher influenza uptake than did larger homes (see table 1) although the opposite was true for pneumococcal vaccine. It seems plausible that smaller homes might find it easier to ensure vaccination of its residents although our study may not be large enough to demonstrate this. We found that homes served by more than 5 GPs had higher pneumococcal vaccine uptake than those served by 5 or fewer (Table 1). As it would be more intuitive to suppose that homes served by fewer practices may have better uptake, this association may be due to chance, or alternatively there may be some other confounding factor involved, such as care home location.

Standards for care homes are currently enforced by the Commission for Social Care Inspection (CSCI), through a system of both planned and ad-hoc inspections. Currently, although their remit does include infection control, information pertaining to vaccine uptake and vaccination policy do not form part of such inspections. In view of the fact that our study and others suggest many long term care facilities still lack adequate policies to ensuring both residents and staff immunity against vaccine preventable diseases, perhaps this should be a consideration [1,5].

Failure to keep adequate records, particularly for pneumococcal vaccine which is required only once in a lifetime, often means that the vaccination status of an individual is unclear. Although vaccination is advocated in the event of uncertain vaccine status [18], and although this may be desirable in improving vaccine uptake, the decision is not always straightforward due to the relatively high incidence of adverse reactions following repeat vaccinations [19]. Although only one third of homes in our study kept a register of pneumococcal vaccination we did not find this to be associated with uptake.

Difficulty in obtaining consent in the care home setting where cognitive impairment is relatively common can be a barrier to vaccine uptake; indeed only 2 of the care homes we surveyed reported that they would vaccinate without expressed consent. Despite the potentially negative impact upon vaccine uptake, the alternative practice of vaccination without consent or by assuming tacit consent is ethically questionable [20,21]. Care home policy should therefore stipulate that consent for vaccinations should be obtained from residents, or their family if necessary, at the time of admission. This may minimise the likelihood that lack of consent will later act as a barrier to vaccination.

This study is, to the best of our knowledge, the first to report the uptake of pneumococcal vaccine in care homes with nursing following the extension of the UK pneumococcal immunisation programme to include people over 65 years of age. It shows that while influenza vaccine rates seem to be improving among care home residents, pneumococcal vaccine uptake remains low. This study also highlights the fact that most care homes in the Nottingham area do not have vaccination policies in place. This study supports the role of such policies and shows a positive association between having such a policy and vaccine uptake in the case of pneumococcal vaccine.

### Limitations of this study

Using self-completed questionnaires may overestimate vaccine uptake as well as other responses likely to reflect favourably on the institution being studied. One study of care homes which went on to carry out a validation study of its results found that as many as 15% of facilities may have over-reported their vaccination rates [22]. As a result our findings may represent a high estimate of vaccination uptake. However, we attempted to reduce the likelihood of over-reporting by emphasising that our results would be treated confidentially.

## Conclusion

Although coverage of seasonal influenza vaccine in care homes with nursing is relatively high, further improvement would be of benefit. Uptake of pneumococcal vaccine remains low and requires attention. Our survey suggests that vaccination policies within the care home sector are positively associated with vaccine uptake, and that too few care homes have such policies in place. We recommend that care homes should have policies to address the vaccination of residents. These should aim to maximise uptake of influenza and pneumococcal vaccine, as well as deal with consent issues and outbreak situations, which may be particularly pertinent in the event of an influenza pandemic. Regulatory agencies in the sector can encourage the development of such policies by incorporating them as a component of inspections.

## Competing interests

The authors declare that they have no competing interests.

## Authors' contributions

AS designed and implemented the study. JC supervised the study design and helped implement the study. RV performed statistical analysis and assisted in preparation of the manuscript. RCBS formulated the initial study hypothesis and provided advice and guidance on study design and implementation. All authors read and approved the final manuscript.

## Pre-publication history

The pre-publication history for this paper can be accessed here:


